# Portal Vein Thrombosis and Splenic Infarction in a COVID-19 Patient

**DOI:** 10.7759/cureus.16843

**Published:** 2021-08-02

**Authors:** Abdul Rehman, Anoop Jose Thoppil, Sara L Wallach

**Affiliations:** 1 Internal Medicine, Jersey Shore University Medical Center/Saint Francis Medical Center Program, Trenton, USA

**Keywords:** hyper coagulopathy, thrombosis, splenic infarcts, therapeutic anticoagulation, covid-19

## Abstract

COVID-19 is a novel viral infection that primarily affects the lungs and runs the gamut from a mild, self-limiting, febrile illness to respiratory failure and death. It manifested as a global pandemic in 2020 and has since claimed millions of lives. Only a few months into this pandemic, it became evident that the viral infection leads to a hypercoagulable state. Anticoagulants became a standard and important part of therapy while d-dimer became a useful test to guide the choice of the anticoagulant (therapeutic vs prophylactic Lovenox). What remains unclear is how viral pneumonia can cause hypercoagulability, especially when it leads to thrombosis in unusual sites such as the portal vein. Another important question that remains unanswered is the duration of anticoagulation after discharge in the outpatient setting. Our case report addresses both these questions with an intriguing patient who presented with abdominal pain as the chief complaint in the absence of any respiratory symptoms whatsoever.

## Introduction

 In the fall of 2019, a novel coronavirus was identified in the Wuhan province of China, which spread rapidly around the globe and was declared a pandemic - COVID-19 - within three months of the early reported cases [1]. More than one year and 3.8 million deaths later, much has been learned about this lethal respiratory virus - including its genome sequence, leading to the development of a number of highly effective, mRNA-based vaccines. However, it remains challenging to identify the asymptomatic carrier. According to one meta-analysis, 15.6% of patients who tested positive for COVID-19 had no symptoms [2]. Another diagnostically challenging aspect of this viral respiratory infection is its atypical, extra-pulmonary presentation such as abdominal pain due to an underlying deep vein thrombosis (DVT). Early in the course of the pandemic, cohort studies and autopsies strongly suggested an association between COVID-19 and coagulopathy [3]. While substantial evidence informs decision-making regarding anticoagulation in the hospitalized patient, the evidence about anticoagulation after discharge is almost nonexistent.

We describe a case of this viral pneumonia that presented without any respiratory symptoms but with abdominal pain instead in a 33-year-old female.

## Case presentation

**Figure 1:**
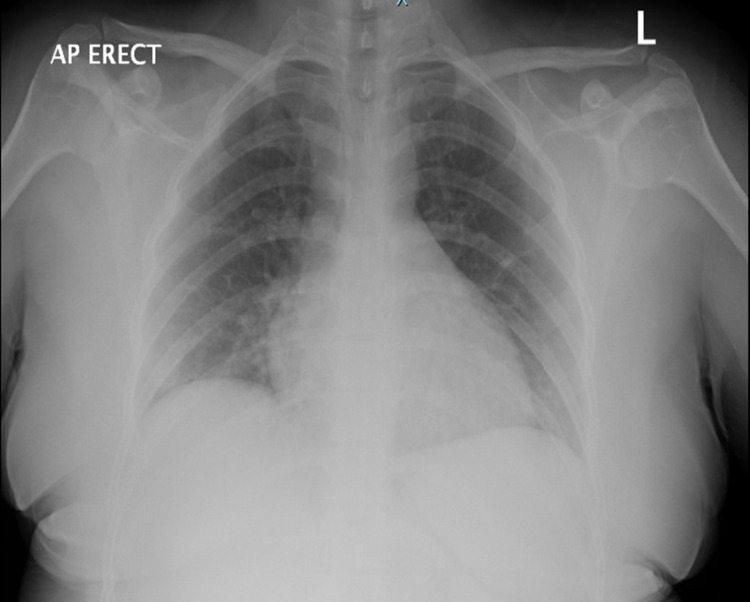
Chest x-ray is negative for ground-glass opacities that are classically present in COVID-19 pneumonia.

**Figure 2:**
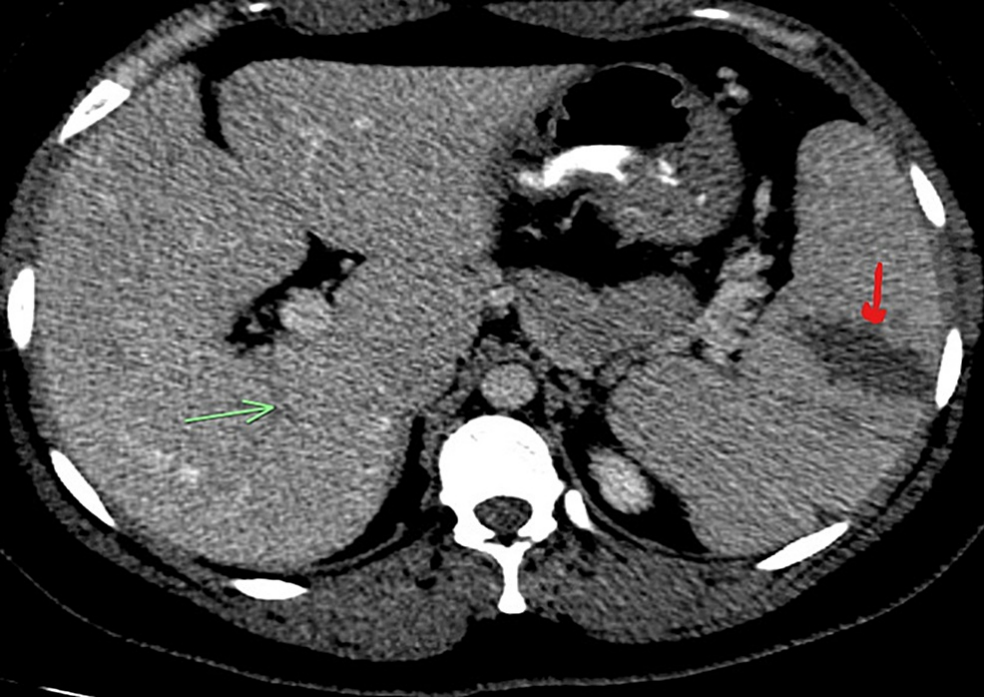
CT abdomen reveals non-opacification of the right portal vein (green arrow). On the right, splenic infarct within the parenchyma is notable (red arrow).

A 33-year-old woman originally from Guatemala with no medical, social or surgical history presented to ED in the summer of 2020 complaining of acute-onset abdominal pain. The patient reported that the pain started earlier in the day and was located in the right lower quadrant (RLQ) with radiation through the umbilicus up to the left upper quadrant (LUQ). It was colicky in character, 7/10 in intensity, with no alleviating or aggravating factors. She denied fevers, cough, shortness of breath, chest pain, diarrhea, constipation or any urinary symptoms. Her BP was 124/68, heart rate was 76/min, the temperature was 38.1 C, respirations were 16/min and oxygen saturation by pulse oximetry was 98% on room air. Physical exam revealed an alert young woman, lying in bed in no apparent distress. Chest exam was clear to auscultation and heart sounds were normal without murmurs (Figure 1), and the abdominal exam was remarkable for mild tenderness in RLQ and LUQ, and normoactive bowel sounds. The neurologic exam was non-focal with intact sensation and reflexes. Basic lab tests showed elevated d-dimer (0.61 µg/mL), fibrinogen (457 mg/dL), C- reactive protein (CRP) (1.45 mg/dL) and erythrocyte sedimentation rate (ESR) (88 mm/h). Liver function tests, lipase, lactate, white cell count, platelet count, LDH, prothrombin time, activated partial thromboplastin time (aPTT), blood urea nitrogen (BUN) and creatinine were all within normal limits. A PA view chest radiograph showed clear lung fields bilaterally. CT abdomen and pelvis with IV contrast revealed non-opacification of the right portal vein - strongly suggestive of thrombosis - as well as a wedge-shaped hypodensity in the spleen, consistent with an acute splenic infarct (Figure 2).

Given the peak of the COVID-19 pandemic and as part of the hospital screening policy, a nasopharyngeal swab for SARS CoV-2 was performed - though there were no respiratory symptoms - and it came back positive. The patient was admitted to the COVID-19 designated floor, started on a therapeutic dose of subcutaneous enoxaparin (1 mg/kg BID) while an extensive autoimmune and genetic workup for hypercoagulability was ordered. The following laboratory tests were ordered: phospholipid B2 glycoprotein antibody, cardiolipin antibodies IgG & IgA, antinuclear antibody, perinuclear anti-neutrophil cytoplasmic antibodies (p-ANCA), cytoplasmic anti-neutrophil cytoplasmic antibodies (c-ANCA), anti-smooth muscle antibody, anti-microsomal antibody, anti-mitochondrial antibody, complement 3 & 4; serologies for hepatitis A, B, C as well as EBV, herpes simplex 1 & 2. All of these tests were negative. On the front of genetic work-up, normal lab assays for factor V Leiden, prothrombin gene as well as normal antithrombin III, protein C & S activities ruled out the more common hereditary hypercoagulable disorders. At this point, by process of elimination, SARS CoV-2 seemed to be the likely culprit for the patient’s unprovoked portal vein thrombosis and splenic infarct.

The patient’s hospital course lasted one week and was uncomplicated. Her abdominal pain continued to improve and resolved on day 8 of hospitalization. Subcutaneous enoxaparin was switched to warfarin upon discharge. The patient was instructed to take this medication for a total of six months and follow up in the INR clinic for dose adjustments.

## Discussion

Although the most concerning symptom of COVID-19 is dyspnea, present in 18.7% of affected individuals, fever and cough are more common symptoms and present in 44% and 68% of affected individuals, respectively [1]. Atypical presentation with abdominal pain or diarrhea is much less common. According to a meta-analysis, 15.6% of infected individuals are asymptomatic [2]. Such patients risk being misdiagnosed and placed on the general medical floor without droplet precautions, posing a serious public health threat. This is particularly a problem in hospitals in the developing world - such as India with its ongoing surge - where they do not have the luxury of testing every patient being admitted. The correlation between COVID-19 and thrombosis is well-documented but poorly understood. Current guidelines that address anticoagulation in hospitalized, COVID-19 patients broadly divide patients into low-risk and high-risk of thromboembolism based on d-dimer levels [3]. However, it remains unclear as to when to taper off anticoagulant therapy in the outpatient setting after the patient has been discharged. Moreover, a review of the literature demonstrates that patients who present with abdominal pain secondary to portal vein thrombosis generally can be retrospectively identified as having symptoms consistent with pneumonia or flu-like symptoms [4-6].

We present this case as an unusual example where the patient could not be identified as having respiratory complaints, fever or "flu-like symptoms” prior to her admission for portal vein thrombosis.

## Conclusions

In our current healthcare climate, the finding of portal vein thrombosis should prompt an investigation for COVID-19 in addition to work-up for hypercoagulability. A patient-tailored approach based on clinical and laboratory data is to be adopted to determine the anticoagulant regimen (prophylactic vs therapeutic heparin). Moreover, the pathogenesis of COVID-19-induced thrombosis does require more study and long-term follow-up of a sizable patient population is needed to determine the duration of anticoagulation. Clinicians should keep COVID-19 on the list of their differential diagnoses when approaching a patient presenting with DVT - even in the absence of any respiratory symptoms.
